# Wirksamkeit der Hörgeräteversorgung bei hochgradigem Hörverlust

**DOI:** 10.1007/s00106-021-01139-5

**Published:** 2022-01-21

**Authors:** Max Engler, Frank Digeser, Ulrich Hoppe

**Affiliations:** Audiologische Abteilung, Hals-Nasen-Ohrenklinik, Kopf- und Halschirurgie, Universitätsklinikum Erlangen, Waldstr. 1, 91054 Erlangen, Deutschland

**Keywords:** Freiburger Sprachtest, Maximales Einsilberverstehen, Sprachaudiometrie, In-situ-Messung, Hochgradiger bis an Taubheit grenzender Hörverlust, Freiburg speech test, Maximum monosyllabic word recognition score, Speech audiometry, Real-ear measurements, Severe to profound hearing loss

## Abstract

**Hintergrund:**

In der Praxis liegt das unilaterale Einsilberverstehen mit Hörgerät bei 65 dB SPL (EV_65_(HG)) häufig unter dem maximalen Einsilberverstehen aus dem Sprachaudiogramm (mEV), insbesondere bei Hörgeräteträgern mit hochgradigem Hörverlust. Diese Arbeit zielte darauf ab, den Wirkungsgrad Q der Hörgeräteversorgung, den Quotienten aus EV_65_(HG) und mEV, bei Hörgeräteträgern mit hochgradigem bis an Taubheit grenzendem Hörverlust zu untersuchen.

**Material und Methoden:**

Es wurden Daten aus In-situ-Messungen, dem Reinton- und Sprachaudiogramm und dem Sprachverstehen mit und ohne Hörgerät von 93 Ohren von 64 Patienten ausgewertet. Die Patienten stellten sich im Jahr 2019 für eine Hörgerätekontrolle in unserem Hörzentrum vor. Es wurden die Abweichung der in-situ gemessenen frequenzabhängigen Ausgangspegelwerte von den Zielvorgaben der präskriptiven Anpassformeln NAL-NL2 und DSL v5.0 analysiert. Für die Parameter EV_65_(HG) und Q wurden jeweils die Spearman-Korrelationskoeffizienten für den Sprachverständlichkeitsindex (SII) berechnet.

**Ergebnisse:**

Bei mehr als 67 % der Hörgeräteeinstellungen stimmten die Ausgangspegelwerte mit den Zielkurven für NAL-NL2 oder DSL v5.0 im Bereich von ±5 dB für Frequenzen von 0,5 bis 4 kHz für 65 dB SPL überein. Trotzdem wurde das mEV mit Hörgerät bei 65 dB SPL nicht erreicht (mittlere Abweichungen: 34,4 %). EV_65_(HG) und Q waren jedoch am besten, wenn Zielwerte für DSL v5.0 bei 65 dB SPL erreicht wurden, was mit einem höheren SII einhergeht.

**Schlussfolgerung:**

Für Hörgeräteträger mit hochgradigem bis an Taubheit grenzendem Hörverlust führen die Anpassformeln NAL-NL2 und DSL v5.0 nicht zu einer solchen Verstärkung, dass bei Alltagssprache von 65 dB SPL das mEV erreicht wird. Es bleibt zu untersuchen, ob alternative Präskriptionen mit besserer Hörbarkeit für Eingangspegel von 50 und 65 dB SPL den Wirkungsgrad der Hörgeräteversorgung verbessern könnten.

## Abstract (en)

**Background and objective:**

In practice, the unilateral monosyllabic speech recognition score with hearing aid (WRS_65_(HA)) is often below the maximum word recognition score with headphones (WRS_max_), in particular for subjects with severe hearing loss. The aim of this study was to evaluate the efficiency factor Q of hearing aid provision, the ratio WRS_65_(HA)/WRS_max_, in patients with severe to profound hearing loss.

**Materials and methods:**

Data from real-ear measurements (REM), pure tone and speech audiogram, and speech recognition with and without hearing aid of 93 ears in 64 patients were examined. The patients visited the authors’ hearing center for hearing aid evaluation in 2019. Deviations of the real-ear measured frequency-dependent output level values from the prescription targets NAL-NL2 and DSL v5.0 were analyzed. Spearman correlation coefficients for the speech intelligibility index (SII) were calculated for the parameters WRS_65_(HA) and Q.

**Results:**

In more than 67% of the hearing aid fittings, output level values matched the target curves of NAL-NL2 or DSL v5.0 in the range of ±5 dB for frequencies from 0.5 to 4 kHz at 65 dB SPL. Nevertheless, WRS_max_ was not achieved with hearing aid at conversational speech levels of 65 dB SPL (mean deviations: 34.4%). However, WRS_65_(HA) and Q were best when target values for DSL v5.0 were achieved at 65 dB SPL, which is associated with a higher SII.

**Conclusion:**

For patients with severe to profound hearing loss, the prescription targets of NAL-NL2 and DSL v5.0 do not provide sufficient amplification for WRS_max_ to be achieved at a normal speech level of 65 dB SPL. It remains to be investigated whether alternative prescriptions with better audibility for input levels of 50 and 65 dB SPL might improve the effectiveness of hearing aid provision.

## Hintergrund

Die Prävalenz von Hörstörungen liegt weltweit bei 20,4 %, wobei die Mehrheit der Menschen nur von einem geringgradigem Hörverlust betroffen ist [25]. Der Anteil der Menschen mit hochgradigem bis an Taubheit grenzendem Hörverlust beträgt 0,8 % an der Gesamtbevölkerung bzw. 4 % an den Menschen mit Hörbeeinträchtigung [25]. Der mittlere Hörverlust, der Mittelwert aus den vier Luftleitungshörschwellen bei 0,5; 1; 2 und 4 kHz („four-frequency pure-tone average“, 4FPTA), liegt in dieser Gruppe bei ≥ 65 dB HL. Für die Prävalenz von Hörstörungen in Deutschland finden sich in der Literatur ähnliche Werte zwischen 16 und 25 %, jeweils abhängig von Alter, Studienumgebung, Definition der Hörminderung und Messmethodik [15, 22, 23]. Da für die meisten Hörstörungen keine kausalen Therapien zur Verfügung stehen, werden in der Regel Hörgeräteversorgungen durchgeführt, um das Sprachverstehen in Ruhe und im Störgeräusch zu verbessern. Insbesondere bei hochgradigem Hörverlust ist die Hörgeräteanpassung eine Herausforderung für den Hörakustiker.

Aus dem Reintonaudiogramm allein lassen sich keine hinreichenden Rückschlüsse auf das mit Hörgerät erreichbare Sprachverstehen ziehen [5, 9, 17]. Um den Versorgungserfolg bewerten zu können, werden als Parameter das über Kopfhörer ermittelte maximale Einsilberverstehen (mEV) aus dem Sprachaudiogramm und das Einsilberverstehen im Freifeld bei einem Schalldruckpegel von 65 dB SPL mit Hörgerät (EV_65_(HG)) und ohne Hörgerät (EV_65_) hinzugezogen. In der Hilfsmittel-Richtlinie (HilfsM-RL) wird ein Hörgewinn von mindestens 20 %-Punkten gegenüber der unversorgten Situation bei 65 dB SPL gefordert [6]. Zusätzlich soll das EV_65_(HG) dem mEV „möglichst nahe“ kommen [6]. Untersuchungen von älteren Hörgeräteträgern über 60 Jahre zeigten zwar einen Gewinn durch die Hörgeräteversorgung in 82 % der Fälle, welcher allerdings in 56 % der Fälle unter den 20 %-Punkten lag [14]. Nach Schorn et al. [19, 20] und Kießling et al. [13] gilt eine Hörgeräteversorgung als angemessen, sofern die Abweichungen höchstens 5 %-Punkte [19, 20] bis 10 %-Punkte [13] betragen. Andere Studien zeigten jedoch, dass das mittlere unilaterale EV_65_(HG) ca. 20 %-Punkte unter dem mEV liegt [5, 9, 17]. Die in der Praxis geforderte Annäherung des EV_65_(HG) an das mEV wird in der unilateralen Situation von 77 [9] bis 81 % [14] der Probanden nicht erreicht. Als Erklärung hierfür wurde bereits der geringe Abstand des Pegels, an dem das maximale Einsilberverstehen erreicht wird (L_mEV_), von der Unbehaglichkeitsschwelle genannt, welcher bei 55 % der untersuchten Ohren unter 10 dB lag [9]. Diese geringe Dynamikreserve führt dazu, dass eine adäquate Verstärkungseinstellung, welche nötig wäre, um zu einem angemessenen Sprachverstehen zu gelangen, nahe an die Unbehaglichkeitsschwelle heranführen würde. Beide Parameter mEV und EV_65_(HG) nehmen mit zunehmendem Hörverlust ab, und bei einem 4FPTA ≥ 60 dB HL zeigt sich zusätzlich eine große interindividuelle Varianz [9]. Der L_mEV_ nimmt mit zunehmendem Hörverlust zu, sodass das mEV nicht nur kleiner ausfällt, sondern auch erst bei höheren Pegeln erreicht wird [9].

Um die Verstärkung von Hörgeräten beurteilen zu können, werden In-situ-Messungen ohne („real-ear unaided response“, REUR) und mit Hörgerät („real-ear aided response“, REAR) durchgeführt. Mit der REUR-Messung wird die Übertragungsfunktion des offenen Gehörgangs ermittelt, indem der Schallpegel am Trommelfell mit einem Sondenmikrofon gemessen wird. Für die Messung der REAR wird der Schallpegel am Trommelfell mit dem platzierten und eingeschalteten Hörgerät gemessen. Die Differenz von REAR und REUR wird als wirksame akustische Verstärkung („real-ear insertion gain“, REIG) bezeichnet. Als Testsignal wird das universelle internationale Sprach-Test-Signal („international speech test signal“, ISTS [8]) verwendet. Mit dieser Vorgehensweise wird der physikalische Einfluss der Anatomie des Außenohrs (Ohrmuschel und Gehörgangsresonanz), der Ankopplung des Hörgeräts an das Ohr (Otoplastik oder Dome) sowie der Art des Schallwandlers (Schallschlauch, externer Hörer oder In-dem-Ohr-Gerät) auf den Pegel des Ausgangssignals mitberücksichtigt. Die gemessene REAR kann anschließend mit theoretischen Zielvorgaben verglichen werden. Die bedeutendsten herstellerunabhängigen Vorschriften zur Bestimmung der Zielwerte sind die beiden präskriptiven Anpassformeln NAL-NL2 („National Acoustic Laboratories-Nonlinear version 2“, [12]) und DSL v5.0 („Desired Sensation Level version 5.0“, [21]). Mit diesen Anpassformeln lassen sich frequenzabhängige Zielkurven für geringe (50 dB SPL), mittlere (65 dB SPL) und hohe (80 dB SPL) Eingangspegel berechnen.

### NAL-NL2

NAL-NL2 wurde mit dem Ziel entwickelt, das Sprachverstehen zu maximieren und dabei die Lautheit auf diejenige von normalhörenden Menschen zu begrenzen [12]. Dafür wurde ein Sprachverständlichkeitsmodell, welches auf dem Sprachverständlichkeitsindex („speech intelligibility index“, SII [1]) basiert, sowie ein Lautheitsmodell nach Moore & Glasberg [16] in einem adaptiven Optimierungsprozess verwendet. Zunächst wird die frequenzabhängige Verstärkung bestimmt, um das Sprachverstehen zu maximieren. Anschließend wird die empfundene Lautheit abgeschätzt und mit der Lautheit verglichen, die ein Mensch ohne Hörbeeinträchtigung in derselben Situation empfinden würde. Liegt die empfundene Lautheit des Hörbeeinträchtigten über der von normalhörenden Menschen, wird die Gesamtverstärkung reduziert. Neben einer binauralen Korrektur berücksichtigt NAL-NL2 unter anderem auch einen möglichen Schallleitungsanteil, die Anzahl der Frequenzkanäle, die Hörgeräteerfahrung und die Ankopplung des Hörgeräts an das Ohr (Otoplastik und Durchmesser der Zusatzbohrung oder Dome).

### DSL v5.0

Das Ziel von DSL v5.0 ist es, möglichst viele für das Sprachverstehen relevante Sprachanteile in den hörbaren Bereich innerhalb der Restdynamik zu bringen, ohne dabei die Unbehaglichkeit zu erreichen (Lautheitsnormalisierung) [3, 21]. Im Gegensatz zu NAL-NL2 wird außerdem die gemessene Unbehaglichkeitsschwelle berücksichtigt.

Der wesentliche Unterschied zwischen den resultierenden Zielkurven der beiden Anpassformeln ist, dass mit steigendem Hörverlust bei DSL v5.0 typischerweise mehr Verstärkung für mittlere und hohe Eingangspegel berechnet wird als bei NAL-NL2 [4, 18].

Im Rahmen der routinemäßig in unserem Hörzentrum durchgeführten Hörgeräteüberprüfungen werden neben den üblichen Sprachverständlichkeitsmessungen auch die oben beschriebenen objektiven In-situ-Messungen durchgeführt. Da die Hörgeräteanpassungen extern von Hörakustikern außerhalb der Klinik durchgeführt werden, ist nicht bekannt, nach welchen Kriterien die Anpassung der Hörgeräte erfolgte. Die standardisierte Vorgehensweise in unserem Hause besteht daher aus dem Vergleich der in-situ gemessenen REAR sowohl mit den Zielkurven gemäß NAL-NL2 als auch mit denen gemäß DSL v5.0.

Kürzlich wurde von Dörfler et al. [5] ein Schätzwert für den sprachaudiometrischen Wirkungsgrad Q der Hörgeräteversorgung eingeführt. Dieser berechnet sich als Quotient aus EV_65_(HG) und mEV (Q = EV_65_(HG)/mEV) und beschreibt, welcher Anteil des mEV mit dem Hörgerät bei 65 dB SPL umgesetzt werden kann. Dörfler et al. berichteten, dass der Wirkungsgrad in vielen Fällen deutlich unter 1 liegt und mit steigendem Hörverlust abfällt.

Ziel der vorliegenden Studie war es, bei Hörgeräteträgern mit hochgradigem bis an Taubheit grenzendem Hörverlust Zusammenhänge zwischen der gemessenen REAR und dem Sprachverstehen mit Hörgerät bzw. dem Wirkungsgrad Q zu beschreiben. Hierzu wurden die Ohren hinsichtlich der Abweichungen der REAR von den Zielkurven gruppiert und für diese der SII mit dem Sprachverstehen mit Hörgerät bzw. dem Wirkungsgrad korreliert.

## Patienten und Methoden

### Patienten

Für diese retrospektive Studie wurden 100 klinisch durchgeführte Routinekontrollen von Hörgeräteträgern analysiert, wobei insgesamt Messungen von 152 Ohren betrachtet wurden. Die Kontrollen fanden in der Zeit von 08/2019 bis 12/2020 in der Erlanger HNO-Klinik statt. Eingeschlossen wurden uni- und bilateral versorgte Hörgeräteträger sowie bimodal versorgte Patienten, welche auf dem Hörgeräteohr einen 4FPTA von 60 dB HL oder mehr aufwiesen. Weitere Einschlusskriterien waren Deutsch als Muttersprache, vollendetes 18. Lebensjahr, eine abgeschlossene technische Anpassung der Hörgeräte beim jeweiligen Hörakustiker und mindestens 3 Monate Hörgeräteerfahrung. Die letzte Überprüfung und Nachjustierung der Hörgeräteeinstellung lag maximal 6 Monate zurück. Die Ausschlusskriterien waren eine mittlere Differenz von Luft- und Knochenleitungsschwellen zwischen 0,5 und 4 kHz von mehr als 5 dB und erkennbare technische Defekte an den Hörgeräten. Es verblieb ein Kollektiv von 64 Patienten mit uni- oder bilateralem, hochgradigem oder an Taubheit grenzendem Hörverlust (33 Frauen, 31 Männer) im Alter von 24–90 Jahren (Mittelwert und Standardabweichung: 65,8 ± 14,8 Jahre). Daraus resultierten 93 Ohren (48 links, 45 rechts) mit einem 4FPTA von 60,0–105,3 dB HL (Mittelwert und Standardabweichung: 72,9 ± 11,6 dB HL).

### Ton- und Sprachaudiometrie

Mittels Reintonaudiometrie wurden die Luftleitungsschwellen für Frequenzen zwischen 0,125 und 0,5 kHz in Oktavschritten und anschließend bis 8 kHz in Halboktavschritten bestimmt. Die Messung der Knochenleitungs- sowie Unbehaglichkeitsschwellen erfolgte für Frequenzen zwischen 0,5 und 4 kHz in Oktavschritten. Die Messungen des Sprachverstehens wurden in Ruhe und in unilateraler Darbietung mit dem Freiburger Einsilbertest [7] durchgeführt. Es erfolgte eine randomisierte Auswahl der Freiburger Listen, wobei die Listen 1, 3, 5, 14 und 15 nicht verwendet wurden [9]. Zunächst wurde das Sprachverstehen bei 65 dB SPL über Kopfhörer ermittelt. Daraufhin wurde der Präsentationspegel in geeigneter Schrittweite (üblicherweise 10 dB, bei geringer Restdynamik 5 dB) solange erhöht, bis ein Einsilberverstehen von 100 % oder der kleinste nicht mehr tolerierbare Pegel (Unbehaglichkeitsschwelle für Sprache) bzw. die Audiometerleistungsgrenze erreicht wurde. Anschließend wurde im freien Schallfeld über Lautsprecher für jede Seite einzeln der Freiburger Einsilbertest in Ruhe ohne und mit Hörgerät durchgeführt. Die Gegenseite wurde dabei regelgerecht maskiert. Die Freifeldmessungen mit Hörgerät erfolgten in der Regel mit zwei Listen zu 20 Wörtern. Alle Messungen wurden mit einem Audiometer vom Typ AT1000 (Fa. Auritec GmbH, Hamburg, Deutschland) durchgeführt. Das maximale Einsilberverstehen (mEV), der Pegel, bei dem das mEV erreicht wird (L_mEV_) [10] sowie das Einsilberverstehen ohne (EV_65_) und mit Hörgerät (EV_65_(HG)) wurden dokumentiert.

### In-situ-Messungen

Über In-situ-Messungen mit der Aurical II (Fa. NATUS GmbH & Co. KG, Trier, Deutschland) wurde das Hörgerät direkt am Patientenohr vermessen. Hierzu wurde das ISTS mit 50, 65 und 80 dB SPL (L_E‑50_, L_E‑65_, L_E‑80_) über 30 s dargeboten und die REAR aufgezeichnet. Aus den digitalen Messprotokollen heraus wurden für jedes Ohr folgende Parameter extrahiert:

1. Die Hör- und Unbehaglichkeitsschwellen wurden gemäß ANSI 3.6-2004 [2] von dB HL in dB SPL umgerechnet, um mit den REAR-Messungen verglichen werden zu können. Die Schwelle in SPL ergibt sich aus der Schwelle in HL mit Berücksichtigung des hörerspezifischen äquivalenten Bezugs-Schwellenschalldruckpegels („reference equivalent threshold sound pressure level“, RETSPL) und der Differenz zwischen dem Schalldruckpegel im Ohr und dem im 6cc-Kuppler, welcher für die Kalibrierung verwendet wird („real-ear to coupler difference“, RECD): Hörschwelle [dB SPL] = Hörschwelle [dB HL] + RECD_6cc_ + RETSPL, [2].
2. Aus den REAR-Messungen wurden für 20 Frequenzen F_n_ in Terzabständen (F_n_ = 0,125 * 2^(^^*n*^^/3)^ kHz, *n* = 0, 1, …, 19) jeweils die Terzpegel für das mittlere Langzeit-Sprach-Spektrum („long-term average speech spectrum“, LTASS), das 30. Perzentil und das 99. Perzentil berechnet.
3. Die Zielkurven für das LTASS (REAR in dB SPL, Testsignal ISTS) wurden gemäß NAL-NL2 und DSL v5.0 für L_E‑50_, L_E‑65_ und L_E‑80_ bestimmt. Die Zielkurvenberechnung basiert auf den Hörschwellen und weiteren Parametern wie z. B. Hörgeräteerfahrung, Otoplastik und vorhandene Zusatzbohrung. Für DSL v5.0 wird außerdem die gemessene Unbehaglichkeitsschwelle berücksichtigt. Bei hochgradigem Hörverlust kann es vorkommen, dass einige Zielwerte unterhalb der Hörschwellen liegen. Dies kann der geringen Restdynamik geschuldet sein und wird in der Praxis vor allem bei hochgradigem Hochtonhörverlust beobachtet [11].
4. Basierend auf den REAR-Messungen wurde der SII für L_E‑50_, L_E‑65_ und L_E‑80_ berechnet. Gemäß ANSI 3.5-1997 [1] wird zur Bestimmung des SII die Hörbarkeit (0 bis 1) für die 18 verwendeten Terzbänder zwischen 0,16 und 8 kHz ermittelt. Zusätzlich erfolgt eine Gewichtung der einzelnen Terzbänder nach Relevanz (0 bis 1) für das jeweils verwendete Sprachmaterial. Durch das Aufsummieren des Produkts dieser Werte über die verschiedenen Frequenzbänder hinweg wird ein einzelner numerischer Index, der SII, erhalten. Der SII kann von 0 bis 1 oder in % angegeben werden. Ein SII von 0 bedeutet also, dass keine Sprachanteile des jeweiligen Sprachmaterials hörbar sind, während ein SII von 1 eine vollständige Hörbarkeit bedeutet.

### Datenanalyse

Die Datenanalyse erfolgte für jedes der 93 Ohren separat. Hierzu wurden die Daten aus dem Ton- und Sprachaudiogramm, den Freifeld- sowie In-situ-Messungen betrachtet. Zusätzlich wurde aus dem EV_65_(HG) und dem mEV der Wirkungsgrad Q bestimmt. Die Analyse des Shapiro-Wilk-Tests zeigte, dass die erhobenen Daten nicht normalverteilt waren. Für die statistischen Vergleiche der Mediane von mehr als zwei unabhängigen Stichproben wurde daher der Kruskal-Wallis-Test verwendet. Die Post-hoc-Analyse erfolgte mittels Dunn-Bonferroni-Tests. Das Signifikanzniveau betrug für alle Tests α = 0,5. Für die Berechnung von Korrelationen fand das Verfahren nach Spearman Anwendung. Signifikante Unterschiede zwischen den Gruppen sind in den Grafiken durch * (*p* < 0,05), ** (*p* < 0,01) oder *** (*p* < 0,001) gekennzeichnet. Die statistischen Tests wurden mit dem Statistikprogramm Statistical Package for Social Sciences (SPSS V24, Fa. IBM Corp., Armonk, NY, USA) durchgeführt und die Abbildungen mit Matlab® R2020b (Fa. Mathworks, Natick, MA, USA) erstellt.

**Abb. 1:**
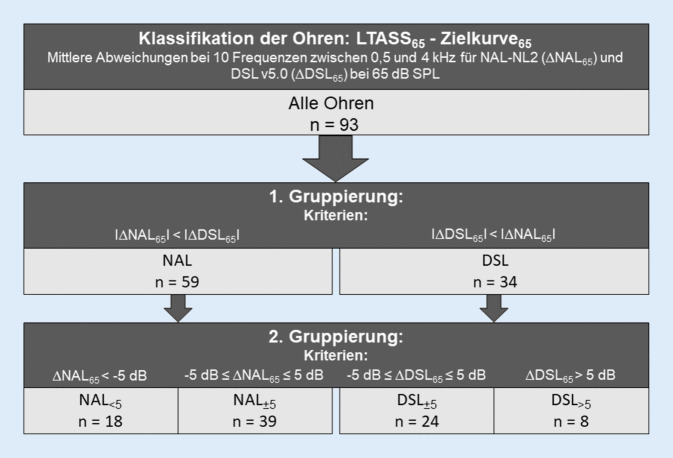
Schematischer Aufbau der Gruppierungsvorgänge. (Abkürzungen siehe Verzeichnis)

Die Daten für die einzelnen Ohren wurden basierend auf den Ergebnissen der In-situ-Messungen gruppiert (Abb. 1). Hierzu wurden bei L_E‑65_ für jedes Ohr die mittleren Differenzen zwischen gemessenem LTASS des Ausgangssignals bei 65 dB SPL (LTASS_65_) und den beiden Zielkurven für die zehn Frequenzen zwischen 0,5 und 4 kHz bestimmt (∆NAL_65_ und ∆DSL_65_). Für |∆NAL_65_| < |∆DSL_65_| wurde das Ohr der NAL-Gruppe und für |∆DSL_65_| < |∆NAL_65_| der DSL-Gruppe zugeordnet. Anschließend wurden die beiden Gruppen unterteilt, in solche mit sehr guter Übereinstimmung an die Zielkurven (NAL_±5_, DSL_±5_) und in solche mit mittleren Abweichungen von mehr als 5 dB von den Zielkurven (NAL_<5_, DSL_>5_).

Für die Parameter EV_65_(HG) und Q wurden jeweils die Spearman-Korrelationskoeffizienten für den SII berechnet. Hierbei ist zu beachten, dass der SII unter Verwendung des ISTS bestimmt wurde. EV_65_(HG) und Q hingegen wurden mittels des Freiburger Einsilbertests bestimmt, welcher eine andere spektrale Verteilung aufweist.

## Ergebnisse

**Abb. 2:**
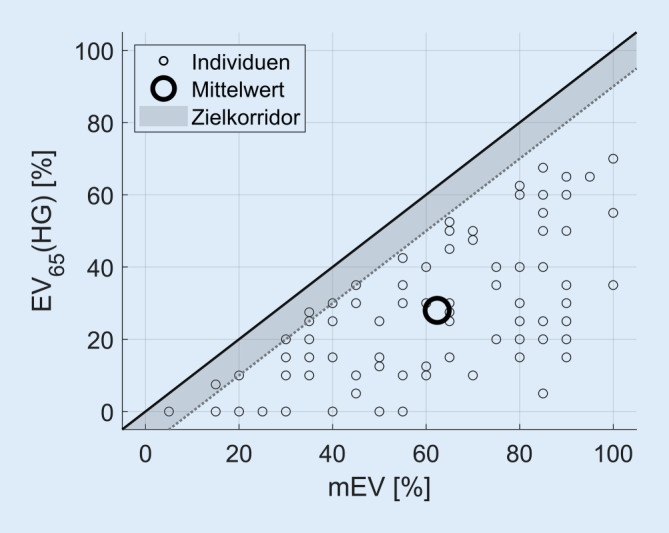
Einsilberverstehen (*n* = 93 Ohren) mit Hörgerät bei 65 dB SPL (EV_65_(HG)) in Abhängigkeit vom maximalen Einsilberverstehen (mEV). *Graue Fläche* Zielkorridor von max. 10 % Diskrepanz zwischen mEV und EV_65_(HG). *SPL *Schalldruckpegel, *dB* Dezibel

In Abb. 2 ist das EV_65_(HG) in Abhängigkeit vom mEV als Scatterplot dargestellt. Die graue Fläche markiert den Zielbereich für eine nach Kießling et al. [13] akzeptable Hörgeräteversorgung. Die Versorgungsergebnisse liegen größtenteils unterhalb des angestrebten Bereichs (91 %). Das mEV wurde in keinem Fall erreicht oder übertroffen. Das mittlere EV_65_(HG) liegt 34,4 %-Punkte unter dem mittleren mEV. Die nach der Hilfsmittel-Richtlinie [6] geforderte Verbesserung von mindestens 20 %-Punkten zeigte sich hingegen bei 65 % der Messungen. Bei 10 % der Hörgeräteversorgungen wurde kein Gewinn erzielt.

### In-situ-Messungen im Vergleich zu den Zielkurven

**Abb. 3:**
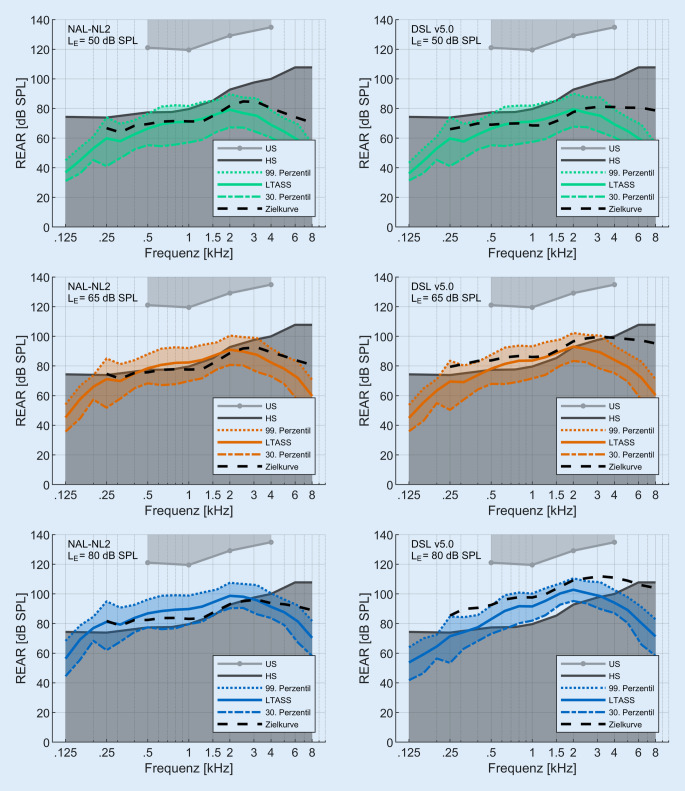
Ausgangsschalldruckpegel am Trommelfell (REAR) als Funktion der Frequenz. Die *obere hellgraue Begrenzung *stellt die Unbehaglichkeitsschwelle (US) und die *untere dunkelgraue Begrenzung* die Hörschwelle (HS) dar. Die *gestrichelte Linie* (*schwarz*) repräsentiert die Zielkurve: NAL-NL‑2 (*linke Spalte*), DSL v5.0 (*rechte Spalte*). Die *durchgezogene farbige Linie* bildet das LTASS, und die *gestrichelten farbigen Linien* bilden das 30. (*untere Linie*) bzw. das 99. (*obere Linie*) Perzentil nach. Die Eingangspegel waren 50 dB SPL (*grün*), 65 dB SPL (*orange*) und 80 dB SPL (*blau*). Gezeigt werden die Mittelwerte über alle Ohren (*n* = 93 Ohren). (Abkürzungen siehe Verzeichnis)

Die Ergebnisse der In-situ-Messungen sind für alle 93 Ohren als Mittelwerte in Abb. 3 für NAL-NL2 in der linken Spalte und für DSL v5.0 in der rechten Spalte für niedrige (L_E‑50_, oben), mittlere (L_E‑65_, mittig) und hohe (L_E‑80_, unten) Eingangspegel dargestellt. Der hörbare, weiß dargestellte Dynamikbereich ist nach unten durch die Hörschwelle und nach oben durch die Unbehaglichkeitsschwelle begrenzt.

- *L*_*E‑50*_*:* Sowohl die Sprachanteile des Ausgangssignals als auch die beiden Zielkurven liegen fast vollständig unter der Hörschwelle. Lediglich die 99. Perzentilkurve liegt im Bereich von 500 bis 1500 Hz leicht über der Hörschwelle.
- *L*_*E‑65*_*:* Das LTASS liegt nur zwischen 0,5 und 2 kHz oberhalb der Hörschwelle. Die Zielkurve für NAL-NL2 und DSL v5.0 unterscheiden sich hier deutlich: Die NAL-NL2-Zielkurve liegt auf oder leicht unterhalb der Hörschwelle, die DSL-v5.0-Zielkurve liegt jedoch um bis zu 12 dB oberhalb der Hörschwelle. Im Mittel liegen alle Zielkurven für DSL v5.0 über den Zielkurven für NAL-NL2 (Mittelwert: 8,8 dB).
- *L*_*E‑80*_*:* Die Zielkurven und das LTASS liegen im Bereich von 0,25 bis 3 kHz auf oder oberhalb der Hörschwelle. Im Mittel liegen alle Zielkurve für DSL v5.0 über diejenigen für NAL-NL2 (Mittelwert: 14 dB).

### Ausgangsschalldruckpegel für die einzelnen Verstärkungsgruppen

**Abb. 4:**
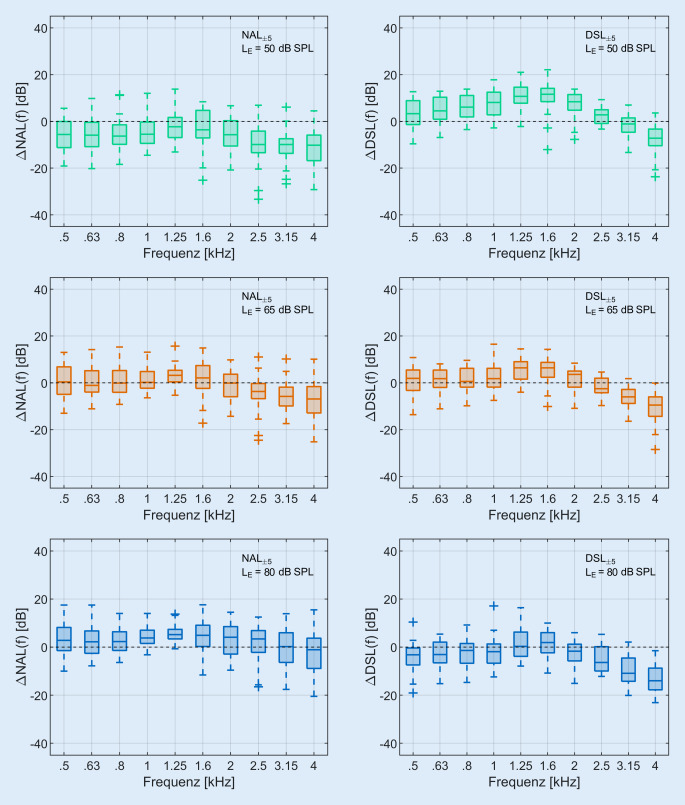
Differenz zwischen LTASS und Zielkurve für NAL-NL2 (∆NAL(f), *n* = 39) in der *linken Spalte* und für DSL v5.0 (∆DSL(f), *n* = 24) in der *rechten Spalte* für NAL_±5_ und DSL_±5_ im Frequenzbereich von 0,5–4 kHz. *Boxplots* Median (*−*), Interquartilsabstand (Boxlänge), Whiskers (maximal das 1,5-Fache des Interquartilsabstands) und Ausreißer (*+*) Die Eingangspegel waren 50 dB SPL (*grün*), 65 dB SPL (*orange*) und 80 dB SPL (*blau*). (Abkürzungen siehe Verzeichnis)

**Abb. 5:**
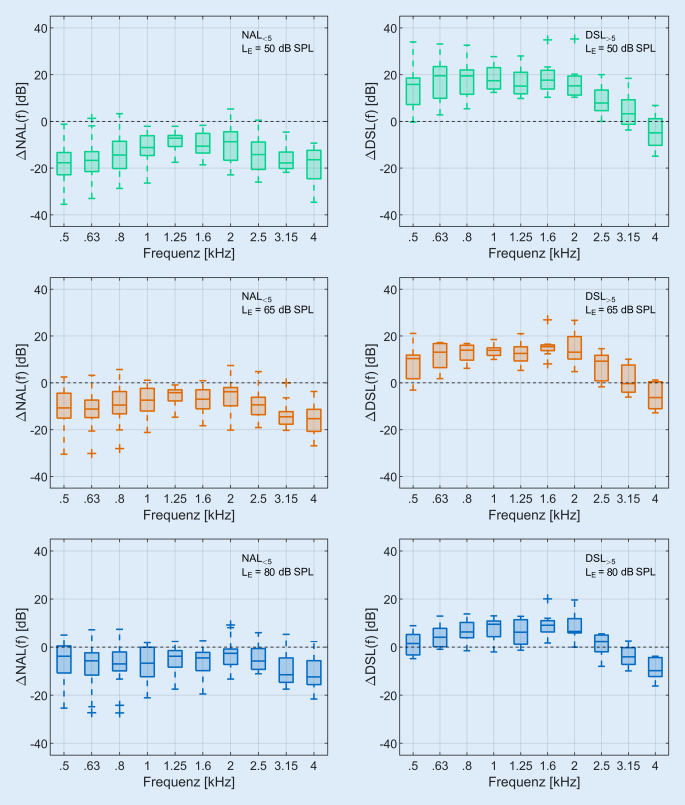
Differenz zwischen LTASS und Zielkurve für NAL-NL2 (∆NAL(f), *n* = 18) in der *linken Spalte* und für DSL v5.0 (∆DSL(f), *n* = 8) in der *rechten Spalte* für NAL_<5_ und DSL_>5_ im Frequenzbereich von 0,5–4 kHz.  *Boxplots* Median (*−*), Interquartilsabstand (Boxlänge), Whiskers (maximal das 1,5-Fache des Interquartilsabstands) und Ausreißer (*+*). Die Eingangspegel waren 50 dB SPL (*grün*), 65 dB SPL (*orange*) und 80 dB SPL (*blau*). (Abkürzungen siehe Verzeichnis)

Aus dem Vergleich von dem LTASS_65_ und den Zielkurven bei L_E‑65_ wurden wie oben beschrieben vier Gruppen gebildet (Abb. 1). Dabei war die Übereinstimmung zwischen Ziel und Messung bei 39 Ohren für NAL-NL2 (NAL_±5_) und bei 24 Ohren für DSL v5.0 (DSL_±5_) am größten. Bei 18 Ohren lag das LTASS_65_ deutlich niedriger als die NAL-NL2-Zielkurve (NAL_<5_). Bei 8 Ohren lag das LTASS_65_ deutlich über der DSL-v5.0-Zielkurve (DSL_>5_). Es verblieben vier Ohren, welche keines der Kriterien aus Abb. 1 erfüllten. Hier lag das LTASS_65_ mehr als 5 dB über NAL-NL2 und mehr als 5 dB unter DSL v5.0. Die Differenzen zwischen LTASS und Zielkurve (∆NAL(f) und ∆DSL(f)) sind in Abb. 4 für die Gruppen mit gut passender REAR (NAL_±5_ links und DSL_±5_ rechts) dargestellt. Die Abb. 5 zeigt die gleiche Ansicht für Gruppen mit deutlich niedrigerer (NAL_<5_), bzw. deutlich höherer REAR (DSL_>5_).

Obwohl das LTASS_65_ und die Zielkurve für die beiden Gruppen NAL_±5_ und DSL_±5_ bei L_E‑65_ sehr gut übereinstimmen, sind bei niedrigeren und höheren Eingangspegeln deutliche Abweichungen zu beobachten. Für L_E‑50_ liegt das LTASS_50_ tendenziell über der Zielkurve für DSL v5.0 und unterhalb derjenigen für NAL-NL2. Bei L_E‑80_ liegt das LTASS_80_ für NAL_±5_ größtenteils oberhalb der Zielkurve und für DSL_±5_ unterhalb.

Die in Abb. 5 dargestellten Differenzen zwischen LTASS und Zielkurve für NAL_<5_ (linke Spalte) und DSL_>5_ (rechte Spalte) zeigen, dass die Abweichungen für alle drei Eingangspegel zu finden sind. Die Abweichungen sind für L_E‑50_ am größten und nehmen mit zunehmendem Eingangspegel ab.

### Einsilberverstehen mit Hörgerät und Wirkungsgrad der Hörgeräteversorgung in Abhängigkeit vom Sprachverständlichkeitsindex

Die vier Gruppen (NAL_<5_, NAL_±5_, DSL_±5_ und DSL_>5_) werden durch unterschiedliche Symbole repräsentiert. Die schwarzen Symbole bilden jeweils die Mittelwerte der einzelnen Gruppen. Zusätzlich werden die vier restlichen Ohren gezeigt, bei denen keines der Gruppierungskriterien erfüllt wurde (Andere). Obwohl große Überschneidungen der Gruppensymbole auftreten, gibt es Unterschiede zwischen den Gruppen hinsichtlich der Parameter EV_65_(HG), Q und SII. In Tab. 1 werden die Mediane und die Mittelwerte der einzelnen Parameter für die vier Gruppen dargestellt. Die Analyse des Kruskal-Wallis-Tests zeigte für 4FPTA, EV_65_(HG), Q und SII einen hochsignifikanten Gruppeneffekt. Die Ergebnisse der Dunn-Bonferroni-Tests sind in Tab. 2 aufgeführt.

**Tab. 1:** Verstärkungsgruppen NAL_<5_, NAL_±5_, DSL_±5_ und DSL_>5_ mit dazugehörigen Median- und Mittelwerten für die einzelnen Parameter

**–**	NAL_<5_		NAL_±5_		DSL_±5_		DSL_>5_	
Anzahl	18		39		24		8	
Min/max ∆NAL_65_/∆DSL_65_ [dB]	−22/−5,3		−4,8/3,9		−4,8/4,7		5,1/14,2	
–	*Median*	*Mittelwert*	*Median*	*Mittelwert*	*Median*	*Mittelwert*	*Median*	*Mittelwert*
Alter [Jahre]	65	65,4	68	65,9	69	65,3	78	70
4FPTA [dB HL]	81,6	81,2	68,3	72,1	67,3	69,3	69,9	71,4
L_mEV_ [dB SPL]	110	108,6	100	105	110	106,3	110	112,5
mEV [%]	57,5	53,6	55	59,7	80	71,7	67,5	65
EV_65_ [%]	0	2,2	0	2,7	0	3,1	0	0
EV_65_(HG) [%]	10	15,4	20	24,5	40	40,6	32,5	31,9
Q	0,36	0,26	0,31	0,39	0,67	0,58	0,48	0,48
SII_65_	0,14	0,15	0,35	0,33	0,48	0,48	0,59	0,57

(Abkürzungen siehe Verzeichnis)

**Tab. 2:** Vergleich der Verstärkungsgruppen NAL_<5_, NAL_±5_, DSL_±5_ und DSL_>5_: Ergebnisse der Kruskal-Wallis-Tests (Gruppeneffekt) und der Dunn-Bonferroni-Tests (Paarvergleich) für die einzelnen Parameter

–	Kruskal-Wallis-Test	NAL_<5_/NAL_±5_	NAL_<5_/DSL_±5_	NAL_<5_/DSL_>5_	NAL_±5_/DSL_±5_	NAL_±5_/DSL_>5_	DSL_±5_/DSL_>5_
Alter	χ^2^(3) = 0,791 *p* = 0,852	–	–	–	–	–	–
4FPTA^*^	χ^2^(3) = 10,711 *p* = 0,013	*p* = 0,084	*p* *=* *0,009*	*p* = 0,144	*p* = 1,000	*p* = 1,000	*p* = 1,000
L_mEV_	χ^2^(3) = 6,332 *p* = 0,097	–	–	–	–	–	–
mEV	χ^2^(3) = 6,856 *p* = 0,077	–	–	–	–	–	–
EV_65_	χ^2^(3) = 3,014 *p* = 0,389	–	–	–	–	–	–
EV_65_(HG)^*^	χ^2^(3) = 20,797 *p* < 0,001	*p* = 0,714	*p* *<* *0,001*	*p* = 0,155	*p* *=* *0,004*	*p* = 1,000	*p* = 1,000
Q^*^	χ^2^(3) = 18,069 *p* < 0,001	*p* = 0,559	*p* *<* *0,001*	*p* = 0,311	*p* *=* *0,014*	*p* = 1,000	*p* = 1,000
SII_65_^*^	χ^2^(3) = 55,115 *p* < 0,001	*p* *=* *0,004*	*p* *<* *0,001*	*p* *<* *0,001*	*p* *<* *0,001*	*p* *=* *0,001*	*p* = 1,000

Signifikante Gruppeneffekte werden durch *Asterisk* und signifikante Unterschiede zwischen den einzelnen Gruppen durch *kursiv* markierte Zellen gekennzeichnet (α = 0,05)

(Abkürzungen siehe Verzeichnis)

**Abb. 6:**
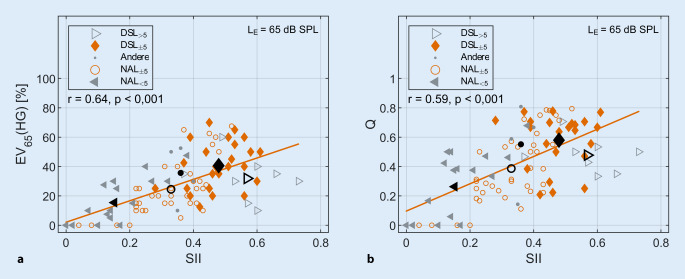
Scatterplot und Korrelationsanalyse (*n* = 93 Ohren). (**a**) Einsilberverstehen mit Hörgerät bei 65 dB SPL (EV_65_(HG)) und Sprachverständlichkeitsindex (SII). (**b**) Wirkungsgrad Q und SII. Zusätzlich sind die Regressionsgeraden (*orange*) eingezeichnet. Den verschiedenen Gruppen wurden unterschiedliche Symbole zugeordnet: NAL_<5_ (*Dreieck, nach links zeigend*), NAL_±5_ (*Kreis*), DSL_±5_ (*Diamant*), DSL_>5_ (*Dreieck, nach rechts zeigend*) und Andere (*Punkt*). Die schwarzen Symbole bilden den Mittelwert der einzelnen Gruppen. (Abkürzungen siehe Verzeichnis)

In Abb. 6 wird der SII jeweils mit dem EV_65_(HG) (linke Spalte) und mit dem Wirkungsgrad Q (rechte Spalte) in Form von Scatterplots für alle 93 Ohren in Beziehung gesetzt. Trotz der großen Variabilität ergibt sich eine hochsignifikante Korrelation von r = 0,64 (*p* < 0,001) für EV_65_(HG) und r = 0,59 (*p* < 0,001) für Q. Der Mittelwert und die Standardabweichung betragen jeweils für EV_65_(HG) 28 ± 19,1 % und für Q 0,43 ± 0,24.

### Einfluss des Schallpegels für das maximale Einsilberverstehen auf das Einsilberverstehen mit Hörgerät und den Wirkungsgrad der Hörgeräteversorgung

**Abb. 7:**
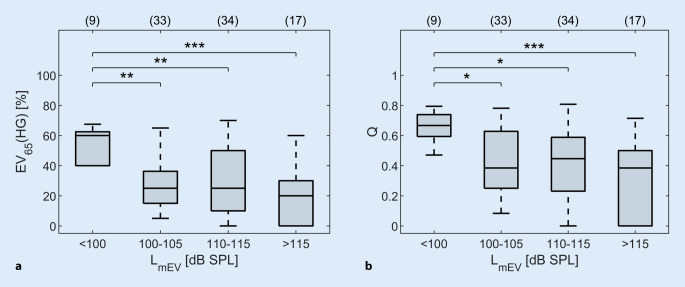
Einsilberverstehen mit Hörgerät bei 65 dB SPL (EV_65_(HG), **a**) und Wirkungsgrad (Q, **b**) in Abhängigkeit vom Pegel für das maximale Einsilberverstehen (L_mEV_). *Boxplots* Median (*−*), Interquartilsabstand (Boxlänge) und Whiskers (maximal das 1,5-Fache des Interquartilsabstands). *Asteriske* In der Post-hoc-Analyse festgestellte Signifikanzniveaus, *p* < 0,05 (*), *p* < 0,01 (**) und *p* < 0,001 (***). *dB* Dezibel, *SPL* Schalldruckpegel

Alle 93 Ohren wurden in vier Gruppen eingeordnet, um den Effekt des Pegels für das maximale Einsilberverstehen (L_mEV_) auf EV_65_(HG) und Q zu untersuchen. In Abb. 7 werden die Ohren in Boxplots wie folgt zusammengefasst: L_mEV_ < 100 dB SPL (G1), L_mEV_ = 100–105 dB SPL (G2), L_mEV_ = 110–115 dB SPL (G3) und L_mEV_ > 115 dB SPL (G4). Die Mediane betragen für EV_65_(HG) 60 % (G1), 25 % (G2, G3) und 20 % (G4) und für Q 0,67 (G1), 0,38 (G2), 0,45 (G3) und 0,38 (G4). Für die beiden Parameter EV_65_(HG) und Q konnte ein signifikanter Gruppeneffekt gefunden werden (Tab. 3). Die Dunn-Bonferroni-Tests ergaben, dass die Patienten in der G1-Gruppe mit einem L_mEV_ von < 100 dB ein signifikant besseres EV_65_(HG) bzw. Q erreichten, als die Patienten in den anderen Gruppen (Tab. 3). Zwischen den Gruppen G2, G3 und G4 gab es keine signifikanten Unterschiede. Für G2 bis G4 ist außerdem eine größere Variabilität der Daten zu erkennen.

**Tab. 3:** Vergleich der Gruppen G1 (L_mEV_ < 100 dB SPL), G2 (L_mEV_ = 100–105 dB SPL), G3 (L_mEV_ = 110–115 dB SPL) und G4 (L_mEV_ > 115 dB SPL): Ergebnisse der Kruskal-Wallis-Tests (Gruppeneffekt) und der Dunn-Bonferroni-Tests (Paarvergleich) für die einzelnen Parameter

–	Kruskal-Wallis-Test	G1/G2	G1/G3	G1/G4	G2/G3	G2/G4	G3/G4
EV_65_(HG)^*^	χ2(3) = 15,394 *p* = 0,002	*p* *=* *0,001*	*p* *=* *0,009*	*p* *=* *0,009*	*p* = 1,000	*p* = 1,000	*p* = 1,000
Q^*^	χ2(3) = 11,428 *p* = 0,010	*p* *=* *0,049*	*p* *=* *0,038*	*p* *=* *0,005*	*p* = 1,000	*p* = 1,000	*p* = 1,000

Signifikante Gruppeneffekte werden durch *Asterisk* und signifikante Unterschiede zwischen den einzelnen Gruppen durch *kursiv* markierte Zellen gekennzeichnet (α = 0,05)

*dB* Dezibel, *SPL* Schalldruckpegel, *L*_*mEV*_ Pegel beim maximalen Verstehen im Freiburger Einsilbertest, *EV*_*65*_*(HG)* Einsilberverstehen in Ruhe bei 65 dB SPL im Freifeld mit Hörgerät, *Q* Wirkungsgrad

## Diskussion

In dieser Arbeit wurden sprachaudiometrische Größen in Bezug zu am Trommelfell anliegenden Sprachpegeln gesetzt. Im Fokus standen dabei das Sprachverstehen mit Hörgerät in Ruhe und dessen Beziehung zum maximalen Einsilberverstehen. Weiterhin wurde untersucht, ob das mit Hörgerät erreichte Sprachverstehen von der Abweichung der Hörgeräteverstärkung zu gängigen Zielvorgaben abhängt. In 63 Fällen (67,7 %) wurden die Zielkurven für NAL-NL2 oder DSL v5.0 bei 65 dB SPL mit Abweichungen von ±5 dB zwar erreicht, dennoch liegt das EV_65_(HG) im Mittel um 34,4 %-Punkte unter dem mEV. Der mittlere Wirkungsgrad Q liegt bei 0,43. Somit beträgt das Sprachverstehen mit Hörgerät im Mittel weniger als die Hälfte des mEV. Dies ist vergleichbar mit den Ergebnissen einer früheren Arbeit [5]. Für die 93 untersuchten Ohren nahmen sowohl Q als auch EV_65_(HG) mit zunehmenden SII zu. Da die Zielkurven der mittleren und hohen Eingangspegel für DSL v5.0 über denjenigen für NAL-NL2 lagen, wurden dementsprechend auch bessere Werte für diejenige Ohren gefunden, bei denen das LTASS_65_ näher an der entsprechenden Zielkurve für DSL v5.0 lag.

### Sprachaudiogramm und Sprachverstehen mit Hörgerät

Im vorliegenden Patientenkollektiv liegt das mEV zwischen 5 und 100 %. Das EV_65_(HG) liegt zwischen 0 und 70 %. Die mittlere Differenz zwischen mEV und EV_65_(HG) beträgt 34,4 %-Punkte. Das sind ca. 15 %-Punkte mehr, als früher für ein großes Kollektiv mit unterschiedlichen Schwerhörigkeitsgraden beschrieben wurde [9]. Bei acht Ohren (8,6 %) liegen diese Differenzen im geforderten Bereich zwischen 5 %-Punkten [19, 20] und 10 %-Punkten [13], allerdings wird das mEV in keinem Fall erreicht oder übertroffen. In der Literatur erfüllten 19 [14] bis 23 % [9] diese Forderungen. Die Vorgabe aus der Hilfsmittel-Richtlinie (Verbesserung um mindestens 20 %-Punkte [6]) wurde in 60 Fällen (64,5 %) erfüllt. Das sind im Vergleich zur Literatur ca. 20 %-Punkte mehr [14]. Die Unterschiede könnten darin begründet sein, dass in dieser Arbeit ausschließlich Hörgeräteträger mit hochgradiger bis an Taubheit grenzendem Hörverlust ausgewertet wurden. Der mittlere Hörverlust liegt mit 72,9 dB HL deutlich höher als in den zitierten Studien [9, 14].

Um den Wirkungsgrad Q zu bestimmen, wurde der Quotient aus den Parametern EV_65_(HG) und mEV gebildet. Dieser gibt den Anteil am mEV an, welcher mit dem Hörgerät bei 65 dB SPL umgesetzt werden kann [5], beinhaltet allerdings keine Informationen über die Verbesserung des Sprachverstehens durch das Hörgerät. In diesem Patientenkollektiv befanden sich allerdings nur Patienten mit einem 4FPTA ≥ 60 dB HL auf dem Hörgeräteohr, und das EV_65_ lag größtenteils bei 0 %. Ein Q > 0 entspricht somit in den meisten Fällen bereits einer Verbesserung durch das Hörgerät.

Für die Bewertung bei einzelnen Patienten ist der Wirkungsgrad Q allerdings kritisch zu interpretieren, da er ein Quotient aus zwei fehlerbehafteten Größen ist. Mittels Fehlerfortpflanzung auf Grundlage der Reliabilitäts-Daten von Winkler et al. 2016 [24] kann die Unsicherheit des Q-Werts geschätzt werden. Für die beiden typischen Werte mEV = 60 % und EV_65_(HG) = 30 % ergibt sich zum Beispiel ein Q‑Wert von 0,5 mit einer hohen Unsicherheit von ±0,38. Für die 93 Ohren liegt diese Unsicherheit im Mittel bei ±0,31. Zur Bewertung des Patientenkollektivs ist der Q‑Wert dennoch geeignet, da aufgrund der relativ großen Anzahl an Messungen die Unsicherheit deutlich kleiner wird. So liegt diese für den mittleren Q‑Wert aller Ohren bei lediglich ±0,03.

### In-situ-Messungen

In Abb. 3 sind die Ergebnisse der In-situ-Messungen für die präskriptiven Anpassformeln NAL-NL2 und DSL v5.0 dargestellt. Für L_E‑50_ ist der Großteil des Sprachsignals für die Patienten unhörbar (Mittelwert und Standardabweichung SII: 0,16 ± 0,12). Der mittlere SII steigt bei L_E‑65_ auf 0,37 ± 0,16 und wird bei L_E‑80_ maximal (0,48 ± 0,16). Für L_E‑65_ und L_E‑80_ liegen im Mittel alle berechneten Zielkurven für DSL v5.0 über den Zielkurven für NAL-NL2. Somit wird für mittlere und hohe Eingangspegel bei DSL v5.0 ein höherer SII erreicht als bei NAL-NL2. Hier stellt sich die Frage, inwieweit die Verstärkung für L_E‑50_ erhöht werden kann, um die Hörbarkeit für leise Sprachanteile wiederherzustellen. Die Maximierung der Hörbarkeit kann dabei nicht völlig unabhängig von den unterschiedlichen Eingangspegeln erfolgen. So lässt sich beispielsweise die Verstärkung für L_E‑50_ meist nicht beliebig erhöhen, sondern nur solange, bis eine ggf. hardwarebedingte, maximale Kompression erreicht wird (z. B. 3:1 für ein bestimmtes Hörgerät). Bei einer weiteren Erhöhung der Verstärkung für L_E‑50_, wird dann automatisch auch die Verstärkung für höhere Eingangspegel erhöht, also in diesem Beispiel für L_E‑65_. Die Verbesserung der Hörbarkeit für L_E‑50_ kann somit auch eine deutliche Erhöhung der Verstärkung für L_E‑65_ und L_E‑80_ mit sich bringen. Daher kommt zusätzlich die Frage auf, wie stark ggf. die Verstärkung für L_E‑65_ und L_E‑80_ erhöht werden kann, ohne die Akzeptanz der Einstellung zu beeinträchtigen. Eventuell kann es sich hier um konkurrierende Ziele in der Hörgeräteanpassung handeln, welche im gegenseitigen Widerspruch stehen, der nicht einfach aufgelöst werden kann. Das Zusammenspiel dieser möglichen Effekte müssten in weiteren Studien systematisch untersucht werden. Bis dahin muss für jeden Hörgeräteträger individuell abgewogen werden, inwieweit entweder eine höhere Verstärkung aller Eingangspegel oder eine Maximierung von L_E‑50_, ohne dabei die Verstärkung für höhere Eingangspegel zu verändern, sinnvoll und umsetzbar ist.

### Hörgeräteevaluierung mittels Parameter aus der Sprachaudiometrie und den In-situ-Messungen

Ein weiteres Ziel der vorliegenden Arbeit war die Untersuchung des Zusammenhangs zwischen EV_65_(HG) bzw. Q und der Annäherung an die präskriptiven Anpassformeln. Die 93 Hörgeräteeinstellungen wurden hierzu den vier Gruppen NAL_<5_, NAL_±5_, DSL_±5_ und DSL_>5_ zugeordnet, entsprechend den mittleren absoluten Abweichungen zwischen LTASS_65_ und der Zielkurve für NAL-NL2 bzw. DSL v5.0 bei 65 dB SPL im Hauptsprachbereich zwischen 0,5 und 4 kHz. Das LTASS_65_ liegt bei 61,3 % der Fälle nahe NAL-NL2 (±5 dB) oder darunter. Bei 34,4 % der Hörgeräteeinstellungen liegt das LTASS_65_ nahe DSL v5.0 (±5 dB) oder darüber. Bei den restlichen 4,3 % fand keine Gruppierung und statistische Analyse statt, da das LTASS_65_ im Bereich zwischen den beiden Zielkurven liegt, aber keines der Gruppierungskriterien erfüllt wurde.

Die Werte der einzelnen Parameter sind für alle vier Gruppen in Tab. 1 dargestellt. In DSL_±5_ waren die Ergebnisse mit Hörgerät, repräsentiert durch die Parameter EV_65_(HG), Q und SII, signifikant höher als in NAL_±5_ und NAL_<5_, obwohl die Voraussetzungen, repräsentiert durch mEV, L_mEV_ und EV_65_, weitgehend gleich waren.

Die Gruppe mit der niedrigsten REAR im Vergleich zu den Zielkurven (NAL_<5_) hatte auch die schlechtesten Hörergebnisse. Allerdings waren auch die Hörverluste in dieser Gruppe mit einem mittleren 4FPTA von 81,2 dB HL am höchsten. Auf der Basis der vorliegenden Daten kann nicht geklärt werden, ob eine größere Verstärkung durch technische oder psychoakustische Gründe verhindert wurde. Die höchsten REARs sind in der Gruppe DSL_>5_ zu finden. Hier liegen auch die SII-Werte deutlich höher. Allerdings ist die höhere REAR und Hörbarkeit nicht mit einem besseren Sprachverstehen verbunden. Im Vergleich zu DSL_±5_ wurden keine signifikant höheren Werte für EV_65_(HG), Q und SII gefunden. Somit ist unklar, ob für eine Verstärkung für L_E‑65_, welche über die Präskription von DSL v5.0 hinausgeht, weitere Verbesserungen in EV_65_(HG) bzw. Q zu erwarten sind. Ob eine Erhöhung der Verstärkung für niedrige Pegel (bzw. eine kompressivere Anpassstrategie) mit einer besseren Annäherung an das mEV einhergeht, bleibt in weiteren Studien zu untersuchen.

Der SII und die Verständlichkeit für Freiburger Einsilber korrelieren signifikant mit 0,64. Höhere Werte sind schon allein deshalb nicht zu erwarten, da die SII-Berechnung durch die kommerzielle Software auf dem ISTS basiert und dementsprechende spezifische Frequenzgewichtungen verwendet. Daraus resultieren Abweichungen, die jedoch weit unterhalb der Bestimmungsfehler für die Sprachverständlichkeit liegen, was a posteriori durch die hohe Korrelation bestätigt wird.

### Grenzen des Versorgungserfolgs

Neben dem maximalen Einsilberverstehen wurde in dieser Arbeit auch der zugehörige Pegel L_mEV_ betrachtet. Für L_mEV_ < 100 dB SPL wurden die besten Ergebnisse erzielt. Die Werte für Q reichen von 0,47–0,79 (Median: 0,67) und streuen vergleichsweise wenig. Für L_mEV_ ≥ 100 dB SPL hingegen streuen die Ergebnisse deutlich mehr und für Q ergaben sich Mediane zwischen 0,39 und 0,45. Dennoch konnten auch bei sehr hohen Werten für L_mEV_ ≥ 115 dB SPL noch vereinzelt relativ hohe Q‑Werte erreicht werden. Statistische Analysen zeigten keine signifikanten Unterschiede zwischen den Gruppen im Bereich von 100 bis > 115 dB SPL.

## Fazit für die Praxis

- Für Hörverluste oberhalb 60 dB HL werden die Zielkurven für NAL-NL2 oder DSL v5.0 bei 65 dB SPL Eingangspegel zwar häufig erreicht, dennoch liegt das Sprachverstehen mit Hörgerät (EV_65_(HG)) oft weit unter dem maximalen Einsilberverstehen (mEV).
- Der Wirkungsgrad Q der Hörgeräteversorgung, berechnet als Quotient aus EV_65_(HG) und mEV, liegt im Mittel bei 0,43 und gibt Auskunft darüber, inwieweit das Potenzial der Hörgeräteversorgung ausgeschöpft wurde.
- Die Hörgeräteeinstellungen, bei denen die Zielkurve für DSL v5.0 bei 65 dB SPL Eingangspegel erreicht wird, führen zu einem besseren EV_65_(HG) und damit auch zu einem besseren Q.

## Abkürzungen

dB: Dezibel
DSL v5.0: Desired Sensation Level version 5.0
DSL_±5_: Gruppierung von Ohren: mittleres LTASS bei 65 dB SPL mit maximalen Abweichungen von ±5 dB von DSL-v5.0-Zielkurve
DSL_>5_: Gruppierung von Ohren: mittleres LTASS bei 65 dB SPL > 5 dB über DSL-v5.0-Zielkurve
EV_65_: Einsilberverstehen in Ruhe bei 65 dB SPL im Freifeld
EV_65_(HG): Einsilberverstehen in Ruhe bei 65 dB SPL im Freifeld mit Hörgerät
HS: Luftleitungshörschwelle
ISTS: Internationales Sprach-Test-Signal („international speech test signal“)
L_E‑50, -65, -80_: Eingangspegel von 50, 65 und 80 dB SPL
L_mEV_: Pegel beim maximalen Verstehen im Freiburger Einsilbertest
LTASS: Mittleres Langzeit-Sprachspektrum („long-term average speech spectrum“)
mEV: Maximales Verstehen im Freiburger Einsilbertest
NAL-NL2: National Acoustic Laboratories-Nonlinear version 2
NAL_<5_: Gruppierung von Ohren: mittleres LTASS bei 65 dB SPL < 5 dB unter NAL-NL2-Zielkurve
NAL_±5_: Gruppierung von Ohren: mittleres LTASS bei 65 dB SPL mit maximalen Abweichungen von ±5 dB von NAL-NL2-Zielkurve
Q: Wirkungsgrad der Hörgeräteversorgung
REAR: Ausgangsschalldruckpegel des Hörgeräts vor dem Trommelfell („real-ear aided response“)
RECD: Differenz zwischen dem Schalldruckpegel im Ohr und dem Schalldruckpegel im Kuppler („real-ear to coupler difference“)
REIG: Verstärkung durch das Hörgerät, REIG = REAR–REUR („real-ear insertion gain“)
REM: Echt-Ohr-Messungen („real-ear Measurements“)
RETSPL: Äquivalenter Bezugs-Schwellenschalldruckpegel („reference equivalent threshold sound pressure level“)
REUR: Schalldruckpegel im unversorgten Ohr vor dem Trommelfell („real-ear unaided response“)
SII: Sprachverständlichkeitsindex („speech intelligibility index“)
SPL: Schalldruckpegel („sound pressure level“)
US: Unbehaglichkeitsschwelle
4FPTA: Mittelwert aus den vier Luftleitungshörschwellen bei 0,5; 1; 2 und 4 kHz („four-frequency pure-tone average“)
∆NAL/∆DSL: Differenz zwischen mittlerem LTASS und mittlerer Zielkurve für NAL-NL2 und DSL v5.0 im Frequenzbereich von 0,5–4 kHz
